# Psychose aigüe secondaire à une dysthyroïdie: à propos de 2 cas

**DOI:** 10.11604/pamj.2016.25.216.10247

**Published:** 2016-12-06

**Authors:** Chadya Aarab, Zakaria Hammani, Rachid Aalouane, Mohsine Yassari, Ismail Rammouz

**Affiliations:** 1 Hôpital Psychiatrique Ibn Alhassan, CHU Hassan II, Laboratoire Neurosciences Cliniques, Faculté de Médecine de Fès, Université Sidi Mohammed Ben Abdellah, Fès, Maroc; 2 Service de Psychiatrie, Hôpital Militaire Moulay Ismail, Meknès, Maroc

**Keywords:** Dysthyroïdie, psychose aigue, hypothyroïdie, hyperthyroïdie, Dysthyroidism, acute psychosis, hypothyroidism, hyperthyroidism

## Abstract

Des anomalies fonctionnelles de la thyroïde sont fréquentes et touchent près de 5 % de la population générale, avec une prédominance pour les femmes. La survenue de troubles mentaux, en particulier de l'humeur, au cours des affections thyroïdiennes est connue depuis plus d'un siècle, alors que les troubles psychotiques associés aux dysthyroïdies sont rarement décrits. Nous rapportons les observations de 2 patientes présentant des troubles psychotiques dans deux situations différentes, la première suite à une hyperthyroïdie avec mauvaise observance thérapeutique et la deuxième secondaire à une hypothyroïdie après une thyroïdectomie. Ces observations suggèrent la responsabilité de l'équilibre hormonal thyroïdien dans l'installation des manifestations psychotiques et elles soulignent la nécessité d'éliminer une dysthyroïdie avant tout traitement psychotrope.

## Abstract (en)

Thyroid function abnormalities are common and affect nearly 5% of the general population, with a female predominance. The occurrence of mental disorders, especially mood disorders, during thyroid diseases is known since a century, while psychotic disorders associated with dysthyroidisms are rarely described. We report 2 cases of patients with psychotic disorders showing two different situations: the first of which following hyperthyroidism with poor medication adherence and the second secondary to hypothyroidism after thyroidectomy. These cases highlight the role for thyroid hormonal balance in the onset of psychotic manifestations and emphasize the need to eliminate dysthyroidism before any psychotropic treatment.

## Introduction

Le dépistage des troubles psychiatriques d'origine secondaire est une préoccupation cruciale pour le psychiatre. Mais comment s'y repérer avec fiabilité parmi un grand nombre d'affections, la plupart d'entre elles ayant une prévalence faible. En pratique courante chaque psychiatre est amené régulièrement à devoir prendre en charge des troubles mentaux de présentation atypique. Ces manifestations peuvent être secondaires à une affection organique générale. Ne pas diagnostiquer ces pathologies entraîne une perte de chance pour le patient, puisque certaines d'entre elles peuvent être prises en charge par des thérapeutiques efficaces [1]. La survenue de troubles mentaux au cours de pathologies endocriniennes est un fait établi depuis plus d'un siècle. Réciproquement, des traumatismes psychologiques peuvent avoir un rôle dans le déclenchement de certaines endocrinopathies, comme dans la maladie de Basedow. Ces dernières années ont vu le développement des examens neuroendocrinologiques chez des patients atteints de troubles psychiatriques indemnes de toute affection endocrinienne, afin d'évaluer l'activité du système limbo-hypothalamo-hypophysaire. Si de nombreuses anomalies neuroendocriniennes ont été décrites au cours des troubles psychiatriques, les méthodologies qui sous-tendent de telles explorations sont loin d'être standardisées. Ainsi, les résultats qui en découlent, parfois contradictoires, se doivent d'être évalués de manière critique. Les tests neuroendocriniens demeurent de précieux outils d'investigation mis à la disposition du clinicien et du chercheur, mais leur pertinence dépend du contexte dans lequel ils sont utilisés [2]. Les dysthyroïdies sont impliquées dans différentes pathologies psychiatriques, surtout dans les troubles de l'humeur. Cependant, les troubles psychotiques sont rarement décrits, pour lesquels seuls quelques cas ont été publiés. Le lien entre ces deux affections est encore mal élucidé. L'objectif de ce travail était de discuter, à travers deux cas cliniques, les troubles psychotiques en lien avec des dysthyroïdies.

## Patient et observation

### Cas 1

Patiente âgée de 53 ans, mariée et mère de 04 enfants, non scolarisée et issue d'un milieu rural. La patiente est sans antécédents psychiatriques, ni médico-chirurgicaux ni toxiques. L'histoire de la maladie remonte à 3 mois avant la consultation en psychiatrie, la patiente ayant consulté en oto-rhino laryngologiste (ORL) pour bilan d'un goitre découvert devant l'apparition d'une masse cervicale, d'un amaigrissement et d'une insomnie. Une échographie cervicale effectuée a révélé un goitre thyroïdien multinodulaire. Les bilans biologiques ont retrouvé un taux de TSH (thyroid stimulating hormone) effondré avec des taux des hormones thyroïdiennes FT3 et FT4 normaux. La recherche d'anticorps antithyroïdiens était négative. Devant ce tableau d'hyperthyroïdie la patiente a été mise sous carbimazole mais avec mauvaise observance. La famille a alors noté progressivement l'apparition d'une irritabilité et d'une insomnie marquée. Par la suite, la patiente a commencé à présenter des états d'agitation répétés, à verbaliser des propos incohérents, un vécu persécutoire, disant que sa fille veut la tuer ou encore que la gendarmerie veut l'arrêter avec une instabilité psychomotrice permanente. Cette évolution s'est faite d'un seul tenant sans retour à l'état prémorbide, et devant l'aggravation progressive de sa symptomatologie, la patiente a été ramenée en consultation psychiatrique par sa famille. L'examen psychiatrique a trouvé une patiente instable sur le plan psychomoteur, avec une angoisse très marquée. L'exploration de la pensée a révélé la présence d'un syndrome délirant mal systématisé, à thème de persécution, à mécanisme intuitif et rapporté avec conviction et forte charge affective, ainsi que la présence d'éléments dissociatifs avec des réponses à côté et des verbigérations. Il n'y avait pas de syndrome hallucinatoire, ni de trouble de l'humeur. Il n'y avait pas non plus de signes de confusion mentale. La patiente était en arrêt de son traitement antithyroïdien qu'elle refusait catégoriquement de prendre, elle a donc été mise sous antipsychotique amisulpride à 200mg/j et prazépam à 20 mg/j. L'évolution a été favorable sous traitement, avec nettoyage complet de sa symptomatologie au bout de 3 mois de traitement. La patiente a ensuite bénéficié d'une thyroïdectomie totale et a été mise sous levothyroxine.

### Cas 2

Patiente âgée de 51 ans, mariée mère de 11 enfants, femme au foyer, de bas niveau socioéconomique, admise aux urgences psychiatriques pour extrême agitation. Dans ses antécédents pathologiques un goitre multinodulaire pour lequel elle a bénéficié d'une thyroïdectomie totale 12 jours avant son hospitalisation, et comme antécédents familiaux deux sœurs psychotiques. L'histoire de la maladie remonte à trois jours après la thyroïdectomie par des cauchemars à répétition suivis au réveil par des propos incohérents et des troubles de comportement avec parfois une agressivité envers ses filles aggravée une semaine après par un refus alimentaire et une insomnie. A l'admission, patiente très agitée avec une bizarrerie comportementale, le contact est difficile, avec des propos incohérents flous et mal systématisés principalement de persécution(ils veulent me tuer; ils veulent me rendre folle). Elle a bénéficié aux urgences somatiques d'un bilan fait d'un scanner cérébral, ionogramme sanguin complet, une numération formule sanguine, un bilan infectieux, rénal et hépatique revenant sans anomalies, tandis que le bilan thyroïdien a révélé une TSHus élevée à 21,066 uUI/ml sachant que le bilan thyroïdien était normal avant l'acte opératoire et la patiente est sous hormonothérapie substitutionnelle après l'opération. La patiente a été hospitalisée à l'établissement psychiatrique, mise sous anxiolytique diazépam 7,5 mg/j, Antipsychotiques Olanzapine 5 mg /j et chlorpromazine200 mg/j. L'évolution a été marquée par une atténuation des symptômes délirants sur une durée de 10 jours. Le suivi en ambulatoire a été caractérisé par une stabilisation de son état et un retour complet à l'état prémorbide avec normalisation de son bilan thyroïdien.

## Discussion

### L'hyperthyroïdie et psychose

Le lien entre thyroïde et symptômes psychiatriques est bien établi. Ainsi, la relation entre troubles de l'humeur et dysthyroïdie est assez bien étudiée, mais les cas de psychose associés à une hyperthyroïdie restent rarement rapportés dans la littérature [3]. Ainsi, à part les cas disparates retrouvés, un seul travail a étudié une série de 18 cas d'hyperthyroïdie associée à des troubles mentaux, afin d'en préciser l'association statistique. Ces cas ont été recrutés sur une période de 20 ans. La moyenne d'âge était de la tranche d'âge de notre patiente (54 ans). Les patients les plus jeunes présentaient une maladie de Basedow et les plus âgés un goitre multinodulaire. L'étude a finalement conclu à l'absence d'un tableau typique de troubles psychiatriques de l'hyperthyroïdie, mais avec une nette prédominance de symptomatologie maniaque et dépressive. Des cas relevant du spectre psychotique, ont été retrouvés en moindre nombre, ce qui souligne la rareté de ce tableau clinique [4], et l'originalité de notre travail. Plusieurs études évoquent l'action synergique des amines biogènes et des hormones thyroïdiennes impliquée dans de nombreux processus métaboliques du cerveau et qui a manifestement un rôle important dans la détermination de l'état psychiatrique. Toutefois, les mécanismes moléculaires et les voies fonctionnelles sous-jacentes à ces effets modulateurs d´hormones sont encore mal cernés [5]. D'un autre côté, d'autres études démontrent une co-occurrence des maladies auto-immunes, les affections inflammatoires chroniques et les troubles mentaux. Dans ces études, une association positive a été trouvée entre la survenue de la maladie de Basedow et troubles psychotiques [6, 7]. Les symptômes neuropsychiatriques régressent habituellement sous traitement antithyroïdien. Cependant, des manifestations psychotiques (nécessitant la prescription de neuroleptiques) ont parfois été rapportées au cours de traitements antithyroïdiens [8]. Les arguments en faveur de l'origine thyroïdienne sont la sévérité et l'ancienneté de l'hyperthyroïdie, le lien chronologique entre les troubles ainsi que l'évolution rapidement favorable des symptômes psychotiques sous traitement antithyroïdien. Ces causes sont dans la plupart des cas curables et de bon pronostic [6].

### L'hypothyroïdie et psychose

L'hypothyroïdie peut être primitive (atrophie thyroïdienne, thyroïdite chronique d'Hashimoto avec goitre qui sont souvent d'origine auto-immune; post-thérapeutique, après traitement à l'iode radioactif ou intervention chirurgicale (c'est le cas de notre patiente) ou secondaire avec baisse de la production de TSH pouvant résulter de la destruction de l'hypophyse et associant généralement des anomalies d'autres hormones hypophysaires, voire tertiaire dont l'origine est un défaut de sécrétion de TRH (Thyreotropin Releasing Hormone) hypothalamique. Lorsque l'hypothyroïdie est d'intensité moyenne, la symptomatologie est dominée par un tableau dépressif associant un ralentissement du débit verbal, une diminution des performances intellectuelles, une fatigabilité, une diminution de l'appétit et une apathie. La « folie myxoedémateuse », dans sa forme la plus sévère, réalise un tableau d'état psychotique confuso-délirant et hallucinatoire; c'est le cas de notre patiente ou d'état mélancolique fréquemment stuporeux, plus rarement d'hypomanie. Chez le sujet âgé, le tableau peut faire évoquer une démence [9]. Une étude transversale menée de juillet à septembre 2013 portant sur 45 patients atteints d'hypothyroïdie a été faite pour préciser la prévalence des troubles psychiatriques chez ces patients et décrire le profil clinique et démographique de ces derniers. L'âge moyen de la population d'étude était de 43 ans avec un sex-ratio de 0,29. 77,7% des patients étaient en hypothyroïdie biologique. Plus du tiers des malades n'ont présenté aucun trouble psychiatrique. Les résultats ont montré que l'hypothyroïdie était associée dans 11,4% des cas à un trouble somatoforme et dans 5,7% des cas à un épisode dépressif majeur. Un trouble obsessionnel compulsif, un trouble panique, un trouble de l'adaptation et un trouble psychotique ont été retrouvés chez 2,8% des cas pour chacun. L'hypothyroïdie était aussi associée dans 42,85% des cas à un trouble anxieux généralisé [10]. En général, les signes physiques et psychiatriques, surtout si l'hypothyroïdie est diagnostiquée précocement, s'améliorent sous traitement hormonal substitutif; cependant, il est estimé que 10 % des patients présentent des symptômes neuropsychiatriques résiduels [9]. Dans ce cas présenté, la patiente a été mise sous traitement hormonal substitutif, et sous antipsychotiques vue la sévérité du tableau clinique, et l'évolution était favorable au bout d'un mois. Chez la plupart des patients ayant une hypothyroïdie, souffrant de troubles psychiatriques, le traitement psychotrope est inefficace tant que l'hypothyroïdie n'est pas corrigée par l'administration de thyroxine. Il est indiqué de pratiquer un bilan thyroïdien en cas de mauvaise réponse à un traitement psychotrope, pour éliminer une éventuelle hypothyroïdie subclinique.

## Conclusion

Ces deux cas cliniques nous rappellent la complexité des interactions entre les hormones et les neurotransmetteurs, ainsi une symptomatologie psychiatrique peut-être la manifestation d'une pathologie endocrinienne, et la dysthyroïdie compliquée de psychose n'est qu'un exemple. Ceci doit être pris en considération dans notre pratique clinique, on doit insister sur l'importance du dépistage et du traitement précoce de l'affection thyroïdienne, de même que sur l'attention à apporter à l'état émotionnel et psychologique du patient atteint de dysthyroïdie.
